# Cytotoxicity and Proapoptotic Effects of* Allium atroviolaceum* Flower Extract by Modulating Cell Cycle Arrest and Caspase-Dependent and* p53*-Independent Pathway in Breast Cancer Cell Lines

**DOI:** 10.1155/2017/1468957

**Published:** 2017-11-08

**Authors:** Somayeh Khazaei, Roslida Abdul Hamid, Vasudevan Ramachandran, Norhaizan Mohd Esa, Ashok Kumar Pandurangan, Fatemeh Danazadeh, Patimah Ismail

**Affiliations:** ^1^Department of Biomedical Science, Faculty of Medicine and Health Sciences, Universiti Putra Malaysia, Selangor, Malaysia; ^2^Malaysian Research Institute of Aging, Universiti Putra Malaysia, Selangor, Malaysia; ^3^Department of Nutrition and Dietetics, Faculty of Medicine and Health Sciences, Universiti Putra Malaysia, Selangor, Malaysia; ^4^School of Life Sciences, B. S. Abdur Rahman Crescent University, Vandalur, Chennai, Tamil Nadu 600048, India

## Abstract

Breast cancer is the second leading cause of cancer death among women and despite significant advances in therapy, it remains a critical health problem worldwide.* Allium atroviolaceum* is an herbaceous plant, with limited information about the therapeutic capability. We aimed to study the anticancer effect of flower extract and the mechanisms of action in MCF-7 and MDA-MB-231. The extract inhibits the proliferation of the cells in a time- and dose-dependent manner. The underlying mechanism involved the stimulation of S and G2/M phase arrest in MCF-7 and S phase arrest in MDA-MB-231 associated with decreased level of* Cdk1*, in a* p53*-independent pathway. Furthermore, the extract induces apoptosis in both cell lines, as indicated by the percentage of sub-G0 population, the morphological changes observed by phase contrast and fluorescent microscopy, and increase in Annexin-V-positive cells. The apoptosis induction was related to downregulation of* Bcl-2* and also likely to be caspase-dependent. Moreover, the combination of the extract and tamoxifen exhibits synergistic effect, suggesting that it can complement current chemotherapy. LC-MS analysis displayed 17 major compounds in the extract which might be responsible for the observed effects. Overall, this study demonstrates the potential applications of* Allium atroviolaceum* extract as an anticancer drug for breast cancer treatment.

## 1. Introduction

After lung cancer, breast cancer is the second leading cause of cancer death among women worldwide. Among more than one million new cancer cases, breast cancer contains 18% of all female cancers globally [1]. Despite significant advances in therapy, breast cancer remains a critical health problem worldwide. Moreover, the current breast cancer treatments are expensive, not widely available, and limited by side effects and resistance to the treatment. Therefore, natural products could be the alternative and novel anticancer agents [2, 3]. Natural crude extracts and biologically active compounds isolated from plant species used in traditional medicine can be prolific resources for new drugs [4].* Allium atroviolaceum (A. atroviolaceum)*, an herbaceous, perennial, bulbous plant, is a species in the genus* Allium* which belongs to the Alliaceae family. It is distributed in Crimea, Caucasus (Ante-Caucasus, Daghestan, and Trans-Caucasus), middle Asia (Mountainous Turkmenistan, Syr-Darya foothill areas), and Iran [5].* A. atroviolaceum* is used as food (vegetable) and a source of vitamins, although useful properties of this species have not been studied adequately [6]. Gas chromatography-mass spectrophotometry study on* A. atroviolaceum* revealed the presence of various bioactive components in the plant extracts which might be the reason of antioxidant and antibacterial potency and perhaps other biological activities [7]. Notably,* Allium* vegetables are rich in flavonols and organosulfur compounds, which have exhibited tumor inhibitory properties in laboratory studies. Some components of* Allium* vegetables are reported to block several stages of carcinogenesis, although the underlying mechanisms of action are generally unclear [8]. The previous studies on* A. atroviolaceum* bulb extract against breast, cervical, and liver cancers [9] and flower extracts against HepG2 [10] and Hela [11] cells demonstrated potential anticancer activity.

In current study methanol extract from flower of* A. atroviolaceum* (FAA) has been used in scientific research to reveal the therapeutic properties of this plant against two human breast cancer cell lines, MCF-7 and MDA-MB-231. We hypothesized that FAA extract induces anticancer effects through the induction of cell cycle arrest and apoptosis in human breast cancer cells. Based on this hypothesis, the study aimed to investigate the possible mechanisms of action in MCF-7 and MDA-MB-231 cells.

## 2. Materials and Methods

### 2.1. Preparation of Plant Extract

The process of extract preparation was described previously [10]. Fresh flowers of* A. atroviolaceum* were collected in July of 2013 from* Kojur*,* Iran* (latitude = 36.3825, longitude = 51.7106, Lat = 36 degrees, 23.0 minutes north, Long = 51 degrees, 42.6 minutes east). The plant was compared with the voucher specimen number 720–722 deposited at the Faculty of Biology Herbarium, Azad University of Ghaemshahr, Iran. Harvested fresh flowers were rinsed and air dried at room temperature. The dried material was homogenized to obtain a coarse powder and extracted by 70% methanol (CheMarCo Inc., SC, USA) for 6 h, using Soxhlet apparatus (Electrothermal Eng., Rochford, UK). The extract was concentrated in a rotary evaporator (Büchi Labortechnik AG, Flawil, Switzerland). The resultant condensate was subjected to freeze-drying (VirTis® BenchTop™ K freeze dryer, NY, USA). The stock solution of extract was prepared by dimethyl sulfoxide (DMSO) (Sigma-Aldrich, MO, USA) prior to usage.

### 2.2. Cell Lines

The human breast cancer cell lines, MCF-7 (human hormone-dependent breast cancer cell line; ATCC HTB-22) and MDA-MB-231 (human non-hormone-dependent breast cancer cell line; ATCC HTB-26), and the normal 3T3 cell line (mouse embryo fibroblast; ATCC CRL-1658) were purchased from American Type Culture Collection (VA, USA). Cell lines were grown in tissue culture flasks at 37°C, 5% CO_2_, and 90% humidity (IR censored CO_2_ incubator) in RPMI-1640 medium (Sigma-Aldrich, Steinheim, Germany), containing 10% fetal bovine serum (Sigma-Aldrich, Steinheim, Germany) and 100 IU/ml penicillin streptomycin (Sigma-Aldrich, Steinheim, Germany). The cells were grown confluence, which could be observed under an inverted microscope and subcultured at three to four days interval.

### 2.3. MTT Cell Proliferation Test

The antiproliferative effects of FAA on MCF-7 and MDA-MB-231 cells were examined using the MTT colorimetric assay. Cells were seeded in 96-well plates (TPP, Switzerland) at a density of 10^6^ cells/ml for 24 h before exposure to the indicated concentrations of FAA for 24, 48, and 72 h. Media without FAA extract were used as a negative control. After removal of the medium, 20 *μ*l of MTT reagent (Sigma-Aldrich, MO, USA) was added to each well and followed by 4 h incubation. The purple formazan crystals were dissolved in 100 *μ*l of DMSO. The optical density (OD) of each well was measured at 570 nm on FLUOstar Omega microplate reader (BMG Labtech, Ortenberg, Germany). An experiment was done in triplicate and cell viability has been calculated by following equation:

(1) $$\begin{array}{c} \%\;\;Cytotoxicity=\frac{\text{OD of treated cells}}{\text{OD of negative control}}\times 100. \end{array}$$

### 2.4. Microscopic Examination

To determine the effects of FAA on the cell morphology, the cells were seeded in 6-well plates (10^6^/ml). After 24 h, 5 ml of media containing IC_50_ concentrations of the FAA was added, while the control contained untreated media. After 24, 48, and 72 h, the cells were then washed with PBS and observed by Olympus Culture Microscope, model CK40 (Olympus Corporation, Tokyo, Japan).

Moreover, acridine orange (AO) and propidium iodide (PI) double staining was applied to observe the apoptotic feature of the cells by fluorescent microscope. MCF-7 and MDA-MB-231 cells were seeded at density of 5 × 10^6^ cells/well and incubated for 24 h; then the medium was replaced with IC_50_ concentration of FAA dissolved in the medium and the cells were incubated for 24, 48, and 72 h. The cell suspension was mixed with an equal volume of AO/PI (Sigma-Aldrich, MO, USA) staining solution (1 : 1). The viable, apoptotic, and necrotic cells were observed under Leica fluorescence microscope DM 2500 (Leica Microsystem, Wetzlar, Germany) within 30 min with 100x magnification. Images were captured by using Alpha Imager (Alpha Innotech, CA, USA).

### 2.5. Flow Cytometric Cell Cycle Analysis

The effect of FAA on MCF-7 and MDA-MB-231 cell cycle distribution was determined by flow cytometric analysis. Cells were seeded at density of 5 × 10^6^, cultured for 24 h prior to treatment with IC_25_, IC_50_, and IC_75_ concentrations of FAA exposure for 24, 48, and 72 h. The cells were then resuspended in 500 *μ*l PBS prior to being fixed at 70% cold ethanol for at least 2 h at –20°C. After centrifuging at 1000 rpm for 10 minutes and washing twice with PBS, the fixed cells were treated with 500 *μ*l PI/RNase (400 *μ*l propidium iodide and 100 *μ*l Ribonuclease A) and incubated for 10 min at room temperature. The DNA content per cell was analysed using BD LSRFortessa™ Cell Analyzer (Becton Dickinson, NJ, USA), equipped with 488 nm argon laser light source and 630 nm band pass filter for 10,000 events per sample. Data was expressed as percentage of cells compared to untreated control population, using along with its analytical software BD FACSDiva™ software.

### 2.6. Annexin-V-FITC/PI

Apoptotic and necrotic cells were differentiated using the Annexin assay V-FITC kit (Sigma-Aldrich, MO, USA) as per the manufacturer's protocol. Cells were plated at 5 × 10^6^ and incubated for 24 h prior to the indication by IC_25_, IC_50_, and IC_75_ concentrations of FAA exposure for 24, 48 and 72 h. The cells were centrifuged (1000 rpm for 5 minutes), resuspended in 1x Annexin-V binding buffer, and stained with 10 *μ*l of PI and 5 *μ*l of Annexin-V-FITC for 10 min in darkness, prior to analyis by flow cytometry. For each experiment 10,000 events per sample were recorded.

### 2.7. Assay of Caspase Activity

Caspase colorimetric protease assay sample kit (Biovision, CA, USA) was used to determine the activity of caspases 1, 2, 3, -5, 6, 8, and 9. Cells (10^6^/ml) were seeded in 6-well plate and incubated for 24 h, prior to exposure to FAA in various concentrations. The cells were then transferred into sterile test tube and lysed using the cell lysis buffer and incubated on ice for 10 min. After centrifugation at 5000 rpm for 2 min, samples (50 *μ*l) of the lysate were aliquoted into a 96-well microplate, to which 50 *μ*l of reaction buffer containing 10 Mm DTT was then added to the sample. Substrates for each of the caspases (5 *μ*l) were added to the appropriate wells and the plate was then incubated for 2 h. Absorbance at 405 nm was then read in a microplate reader. The absorbance of treated samples was compared with untreated control. The selective substrates used were p-nitroaniline.

### 2.8. qPCR Analysis of Cell Cycle and Apoptosis Responsive Genes

Gene's expression was analysed with quantitative reverse transcription-PCR using a real-time SYBR Green gene expression assay kit as described in the protocol. Total RNA was isolated from the MCF-7 and MDA-MB-231 cells, treated with IC_25_, IC_50_, and IC_75_ concentration of FAA for 24 h using RNeasy Mini kit (Qiagen Inc., CA, USA). RNA samples were quantitated at OD 260/280 by NanoDrop Spectrophotometer along with its analytical software V3.7 (Thermo Fisher Scientific, DE, USA) and RIN number was determined by Bioanalyzer (Agilent 2100 Bioanalyzer™ system-Agilent Technologies, Waldbronn, Germany). The RNA (1 *μ*g) was introduced to reverse transcription with RT2 first strand kit (Qiagen Inc., CA, USA) and the cDNA were analysed using RT2 SYBR® Green ROX™ FAST Mastermix (Qiagen Inc., CA, USA). qPCR was performed in a reaction volume of 25 *μ*L as per the manufacturer's instructions. Briefly, 12.5 *μ*L of RT2 SYBR® Green Mastermix, 1 *μ*L of assay primers (RT^2^ qPCR Primer Assays, Qiagen Inc., CA, USA), and 1 *μ*L of template cDNA were added to each well. The PCR plate was placed in the real-time PCR instrument, Corbett Rotor-Gene 6000 (Qiagen Inc., CA, USA), and run at 95°C for 10 minutes (activating the enzyme) and 40 cycles of 15 seconds at 95°C (denaturation) followed by 30 seconds at 60°C (annealing and synthesis). The expression levels were expressed as *n*-fold differences relative to the reference gene, GAPDH. The data were analysed using a comparative threshold (Ct) method, and the fold inductions of samples were compared with the untreated samples. The gene expression level was then calculated by following formula:

(2) $$\begin{array}{c} 2^{\Delta \Delta Ct}=2^{Ct\;\left(treated\;\;cells\right)\;-\;Ct\;\left(control\;\;cells\right)}, \end{array}$$

where 2 is the amplification efficiency where the template doubles in each cycle during exponential amplification.

The following primer pairs for target genes and GAPDH were chosen from the Primer Bank website:* Bcl-2*: 5′-TAC CTG AAC CGG CAC CTG-3′ and 5′- GCC GTA CAG TTC CAC AAA GG-3′;* Cdk1*: 5′- GGGTCAGCTCGCTACTCAAC-3′ and 5′-AAGTTTTTGACGTGGGATGC-3′;* p53*: 5′- TGT GGA GTA TTT GGA TGA CA-3′ and 5′- GAA CAT GAG TTT TTT ATG GC-3′; GAPDH: 5′-TCCTGCACCA CCAACTGCTTAG-3′ and 5′- GGCATGGACTGTGG TCATGAGT-3′.

### 2.9. Evaluation of TAM and FAA Combination Effect

To assess the synergistic effect of FAA extracts in combination with TAM, the cells were seeded at 1 × 10^6^ cells/ml and incubated for 24 h. After incubation, the cells were treated with combination of IC_50_ values of FAA and TAM (obtained in our previous study [9]) and incubated for an additional 24, 48, and 72 h. The MTT solution (20 *μ*l) was added to each well and incubated for 4 h. The MTT-formazan crystals formed were dissolved in 100 *μ*l of DMSO and the absorbance was measured at 550 nm. The IC_50_ obtained for single-agents were compared to the IC_50_ calculated for their combination. The combination index (CI) was calculated by CompuSyn software (Paramus, NJ, USA) to analyse synergistic inhibitory effect. The CI values <1, =1, and >1 represent a synergistic, additive, and antagonistic effects, respectively [12].

### 2.10. Liquid Chromatography-Mass Spectrometry (LC-MS)

LC-MS was used to identify the major compounds in the FAA extract. Chromatographic analysis of FAA was carried out by reverse phase elution (Waters Symmetry LC-18 column 250 × 4.6 mm, 5 *μ*m) on Agilent 6500 Series Accurate-Mass Quadrupole Time-of-Flight (Q-TOF; Agilent Santa Clara, CA, USA) LC/MS system with Agilent 1200 Series Diode Array Detector (module G1315B; detection type: 1024-element photodiode array; light source: deuterium and tungsten lamps; wavelength range 190–950 nm). The mobile phase consisted of (A) formic acid (0.1%, v/v); (B) acetonitrile + 0.1% formic acid; gradient (in solvent B): (i) 20%, from 0 to 20 min, (ii) 95%, from 20 to 27 min, and (iii) 35%, at 27–30 min of total run time; flow rate: 0.2 ml/min; injection volume 3 L; ESI parameters: both negative and positive ion mode; mass range 100–1200 *m*/*z*; spray voltage 4 kV; gas temperature 325°C; gas flow 10 L/min; Nebulizer 40 psi and the mass was analysed by using Agilent technologies Mass-Hunter software.

### 2.11. Statistical Analysis

Results were analysed by version 7 of GraphPad Prism, using one-way analysis of variance (ANOVA), and differences were considered statistically significant at the level of *p* values ≤ 0.05, 0.01, and 0.001. Gene expression analysis was done using Student's *t*-test and the differences were considered statistically significant at the level of *p* values ≤ 0.05.

## 3. Results

### 3.1. Cell Proliferation Inhibition after FAA Treatment

**Figure 1:**
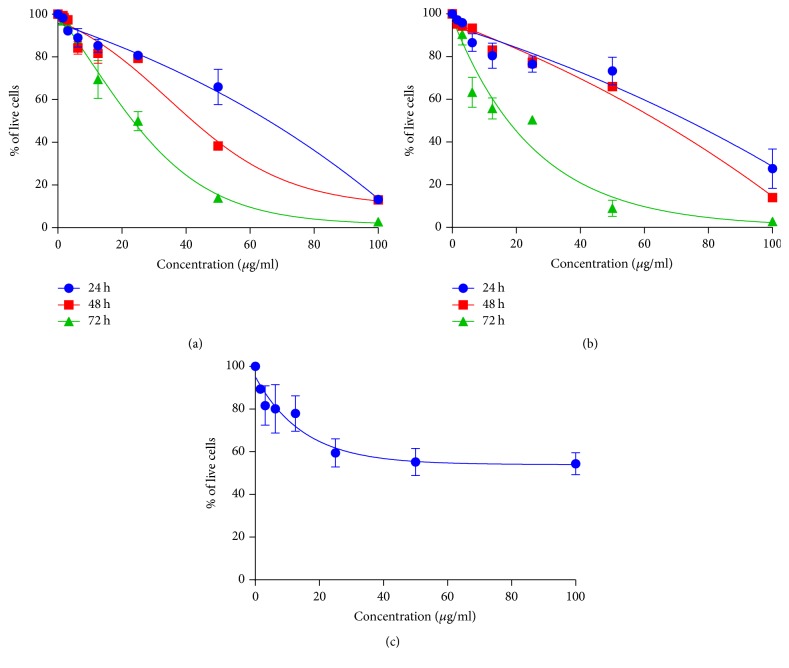
*Antiproliferative effect of FAA on breast cancer and 3T3 cells*. Dose-response curves of FAA treatment on MCF-7 (a), MDA-MB-231 (b), and 3T3 (c) [10]. Data represent the mean ± SD of three independent experiments.

The proliferation of MCF-7 and MDA-MB-231 cells, treated with various concentrations of FAA, was significantly inhibited in a dose- and time-dependent manner. The estimated IC_50_ value was 65.5 ± 9 *μ*g/ml, 41 ± 0 *μ*g/ml, and 24.67 ± 3.5 *μ*g/ml in the case of MCF-7 (Figure 1(a)) and 77 ± 5.29 *μ*g/ml, 67 ± 0 *μ*g/ml, and 51 ± 1.7 *μ*g/ml for MDA-MB-231 cell lines (Figure 1(b)) after 24, 48, and 72 h, respectively. However, based on our previous study FAA showed no evident cytotoxicity against the healthy mouse fibroblast 3T3 cells after 72 h (Figure 1(c)) [10].

### 3.2. Morphological Feature of Apoptosis

The morphological appearances of MCF-7 and MDA-MB-231 after 24, 48, and 72 h of exposure to FAA were observed under the inverted light microscope. In the case of untreated cells, high density of monolayer cells with intact membrane was observed. However, the cells showed morphological changes such as autophagy and reduction in the cell volume after the treatment with IC_50_ concentration of FAA extracts at 24 h. The features of apoptosis were clearly observed after 48 h of treatment, where the treated cells showed the formation of shrinking, membrane blebbing, and membrane bounded vesicles (apoptotic bodies). The extracts showed more cytotoxic effects after 72 h of incubation, since only debris was present in the culture.

**Figure 2:**
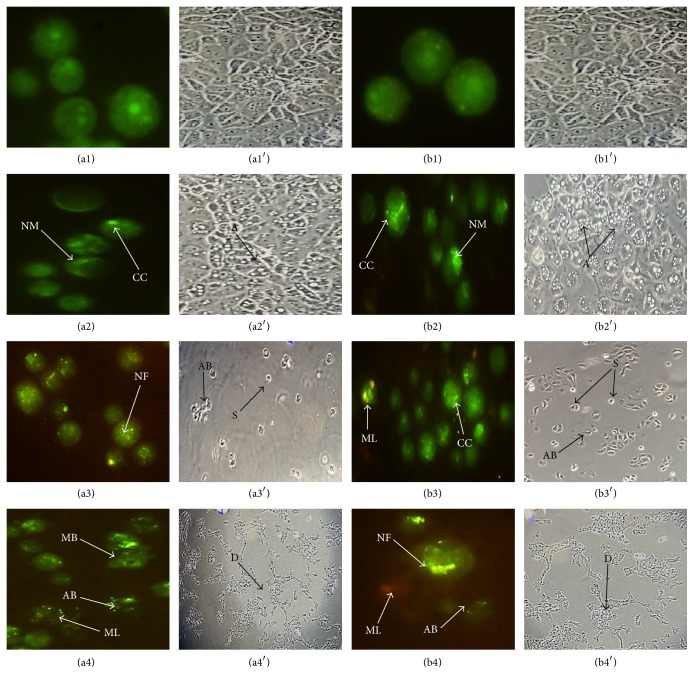
*Morphological observation of treated MCF7 and MDA-MB-231 cells by phase contrast and fluorescence microscopy*. (a1)–(a4) and (a1′)–(a4′) indicate the untreated and treated MCF7 cells with FAA for 24 h, 48 h, and 72 h, respectively, while (b1)–(b4) and (b1′)–(b4′) display the untreated and treated MDA-MB-231 cells with FAA for 24 h, 48 h, and 72 h, respectively. In phase contrast images autophagy (A), shrinkage (S), apoptotic bodies (AB), and debris (D) are shown. In fluorescent images the typical characteristics of apoptosis such as nuclear margination (NM), chromatin condensation (CC), nuclear fragmentation (NF), membrane blebbing (MB), apoptotic bodies (AB), and membrane looseness (ML) are presented. Similar cellular morphology was observed in three independent experiments (*n* = 3).

The morphological changes in the cancer cells nuclei were observed by fluorescence microscope to understand the mode of cell death. AO and PI were used to differentiate between viable, apoptotic, and necrotic cell, based on membrane integrity. The results revealed that FAA triggered morphological features that relate to apoptosis in a time-dependent manner. The untreated cells showed an intact round nuclear membrane with the nuclei heavily stained by AO green fluorescence. After the treatment of MCF-7 with FAA at 24 h, the cell showed typical morphological features of apoptosis, including nuclear margination and chromatin condensation. After 48 h, granulation in the nucleus was clearly observed. The treatment at 72 h with FAA showed membrane blebbing, apoptotic bodies, and membrane looseness. Moreover, similarly to MCF-7, MDA-MB-231 cells exhibited nuclear margination and chromatin condensation, which were major consequences of the apoptotic trigger, after the treatment with FAA at 24 h, while the 48 and 72 h of treatments represented both early (chromatin condensation, nuclear fragmentation) and late apoptosis (apoptotic bodies, membrane looseness) features (Figure 2).

### 3.3. Cell Cycle Arrest in MCF-7 and MDA-MB-231 Cells

**Figure 3:**
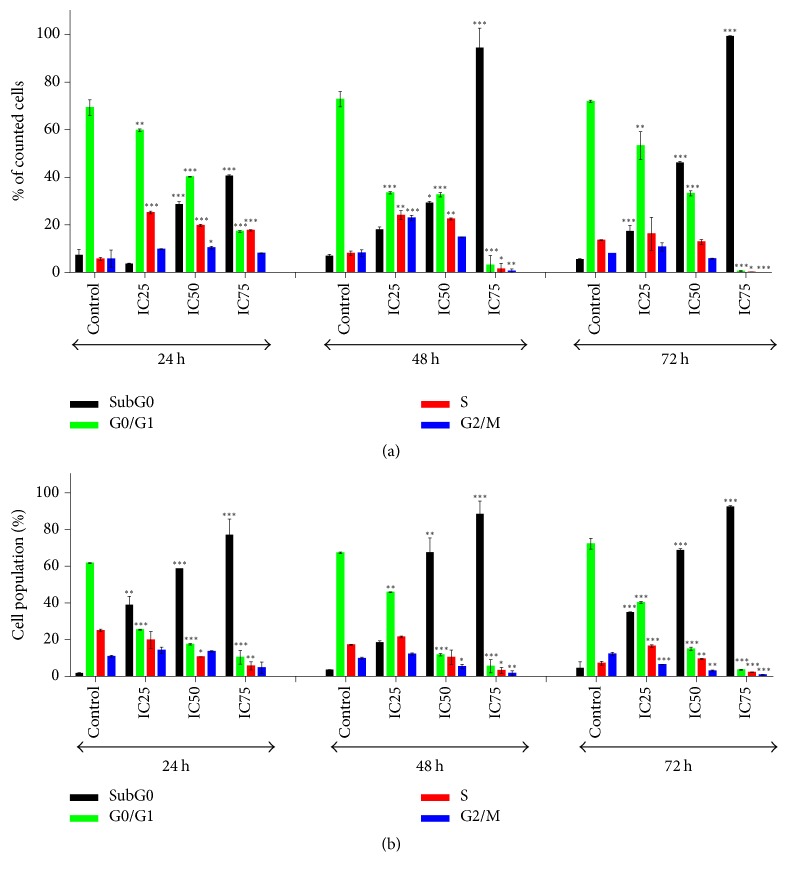
*Cell cycle analysis of MCF7 and MDA-MB-231 cancer cells treated with FAA at IC25, IC50, and IC75 values*. The distribution of (a) MCF7 and (b) MDA-MB-231 cells undergoing apoptosis and in various phases of the cell cycle was determined for 24, 48, and 72 h in comparison to the respective controls. The values are presented as mean ± standard error of mean of three determinations, indicated by *∗*, *∗∗*, and *∗∗∗*, showing a significant difference (*p* < 0.05, *p* < 0.01, and *p* < 0.001, resp.).

The DNA content was measured by flow cytometry to evaluate the cell cycle distribution of MCF-7 and MDA-MB-231 cells with or without FAA treatment. Treatment of MCF-7 presented a subcellular peak and a dramatic decrease in G_0_/G_1_ phase in a dose- and time-dependent manner. At 24 h, distribution of cells in the S phase significantly increments in treated cells with IC_25_, IC_50_, and IC_75_ of FAA, while, after 48 h, this value is considerably increased by IC_25_ and IC_50_ concentrations of FAA. However, the 72 h treatment increased the S phase percentage only in IC_25_ (Figure 3(a), see Figure SI in Supplementary Material available online at https://doi.org/10.1155/2017/1468957). Moreover, 24 h treatment by IC_25_, IC_50_, and IC_75_ of FAA represented a significant enhance of MDA-MB-231 cell distribution at G_2_/M phase. Though, 48 h treatment increased this value by IC_25_ and IC_50_ values of FAA. The event occurred after 72 h of treatment with IC_25_ and IC_50_ of FAA, when the cells were accumulated at the S phase, accompanied by a large enhancement of sub-G_0_ and remarkable reduction of G_0_/G_1_ phase in a time and dose course manner (Figures 3(b), SII).

### 3.4. Induction of Apoptosis in MCF-7 and MDA-MB-231

**Figure 4:**
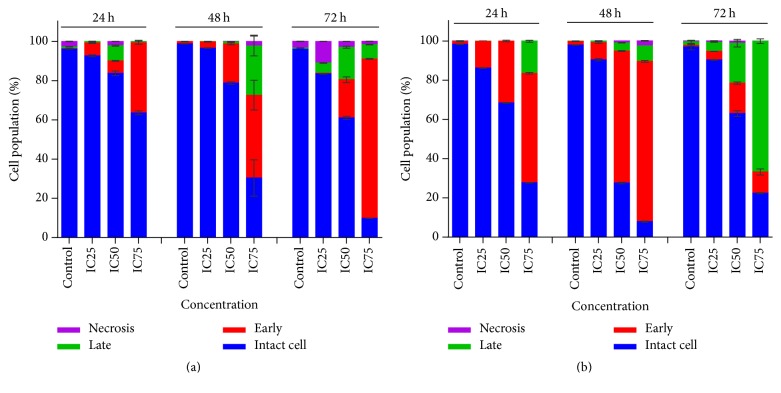
*Apoptosis study of MCF-7 and MDA-MB-231 cells exposed to FAA extract at IC25, IC50, and IC75 values*. The distribution of live cells and cells undergoing early apoptosis, late apoptosis, and necrosis was determined in (a) MCF7 and (b) MDA-MB-231 cells following treatment with FAA for 24, 48, and 72 h in comparison to the respective control, using Annexin-V-FITC and propidium iodide flow cytometric analysis. The values are presented as mean ± standard error of mean of three determinations.

To determine the effect of FAA in apoptosis induction, the FITC-Annexin-V staining was used through flow cytometry. The percentages of live cell significantly declined in both cell lines, after the treatment with FAA in a dose-dependent manner. Analysis of MCF-7 cells showed variation in apoptotic and necrotic cell percentage in time- and dose-dependent manner. The percentage of early apoptotic cells increased after 24 h by IC_25_, IC_50_, and IC_75_, with IC_50_ and IC_75_ values after 48 h and with IC_50_ and IC_75_ of FAA after 72 h. Meanwhile, late apoptosis increased markedly in cells treated with IC_50_ after 24 h, with IC_75_ after 48 h and with IC_25_, IC_50_, and IC_75_ after 72 h. In contrast, the percentages of cells entering necrosis decreased with IC_25_ and IC_75_ of FAA after 24 h and with IC_50_ and IC_75_ after 72 h. No reportable change in the percentage of necrotic cells was observed after the treatment with FAA for 48 h (Figure 4(a)).

The proportion of early apoptotic MDA-MB-23 cells enhanced after 24 h, 48 h, and 72 h with IC_25_, IC_50_, and IC_75_ concentrations of FAA. On the other hand, the cell proportion in late apoptosis was significantly raised in cells treated with IC_75_ of FAA after 24 h, 48 h, and 72 h with IC_25_, IC_50_ and IC_75_. Percentages of cells entering necrosis were significantly enhanced only after 48 h in cells treated with IC_50_ and IC_75_ of FAA (Figure 4(b)).

### 3.5. FAA Treatment Activates the Caspases

**Figure 5:**
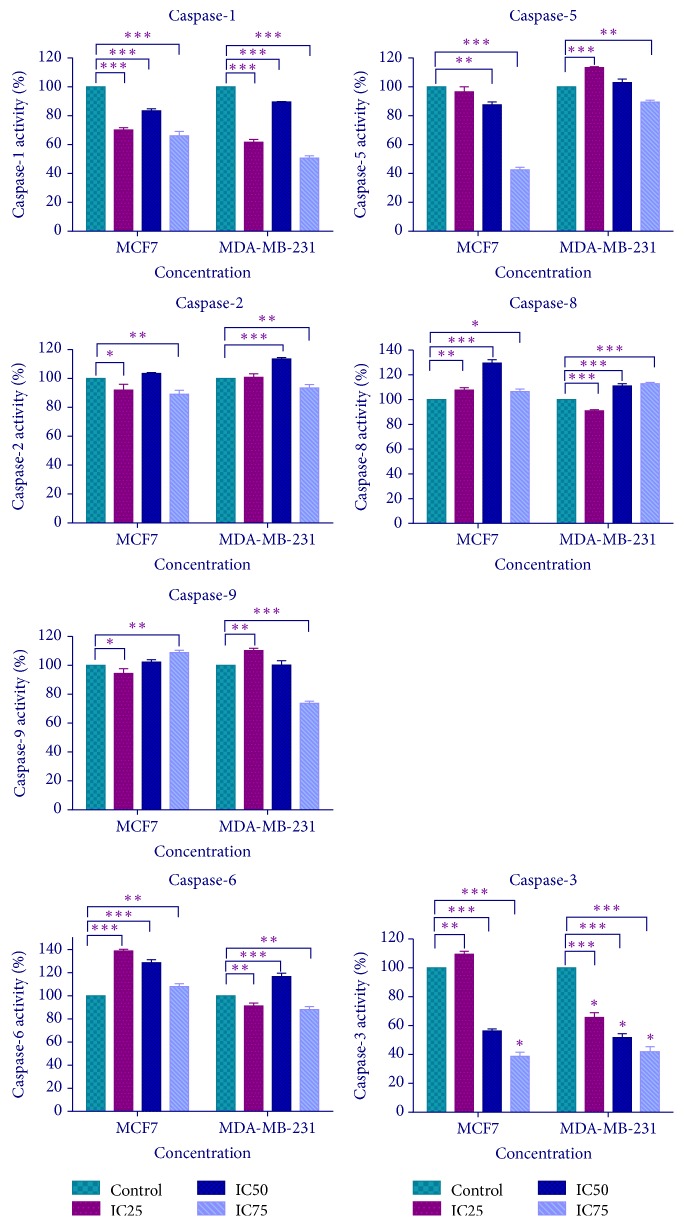
*Activation of caspases by FAA in MCF-7 and MDA-MB-231 cells*. The cells were incubated with FAA extract at IC25, IC50, and IC75 values for 24 h, after which the activity of various caspases was determined as described in Section 2. Data are presented in mean ± SD (*n* = 3). Values for the treated group are significantly different to the untreated control group at the same time point at ^*∗*^*p* < 0.05, ^*∗∗*^*p* < 0.01, and ^*∗∗∗*^*p* < 0.001.

The activation of two inflammatory caspases (caspases 1 and 5), three initiator caspases (caspases 2, 8, and 9), and two executioner caspase (caspases 3 and 6) was investigated at three concentrations of FAA extract in 24 h. Caspase-1 activity in both treated cells resulted in a significant reduction, compared to the untreated cells. The FAA treatment was found to decrease the activity of caspase-5 in MCF-7, while in MDA-MB-231 cells it increased at IC_25_ of FAA. The induction of caspase-2 activity increased significantly in MDA-MB-231, but not in MCF-7. The level of caspase-9 was evaluated significantly at IC_75_ of FAA in MCF-7. In contrast, MDA-MB-231 cells exposed to low concentration (IC_25_) of FAA exhibited a significantly higher activation compared to the control. FAA could trigger caspase-8 activity in both MCF-7 and MDA-MB-231 cell lines in IC_50_ and IC_75_ concentrations. Since both upstream signalling pathways (intrinsic and extrinsic pathway) converge downstream to activate the final effector caspase mechanism, the activity of caspase-3 and caspase-6 executioner caspases was investigated. The results demonstrated that FAA sharply induced caspase-6 activity in MCF-7 cells, as the activity significantly increased in all used concentrations. On the other hand, caspase-6 activity was also enhanced with IC_50_ of FAA in the MDA-MB-231. The FAA extract was found to increase the activity of caspase-3 significantly only in MCF-7 cell line in IC_25_ value (Figure 5).

### 3.6. Expression Levels of* Bcl-2*,* Cdk1*, and* p53* in MCF-7 and MDA-MB-231 Cells

**Figure 6:**
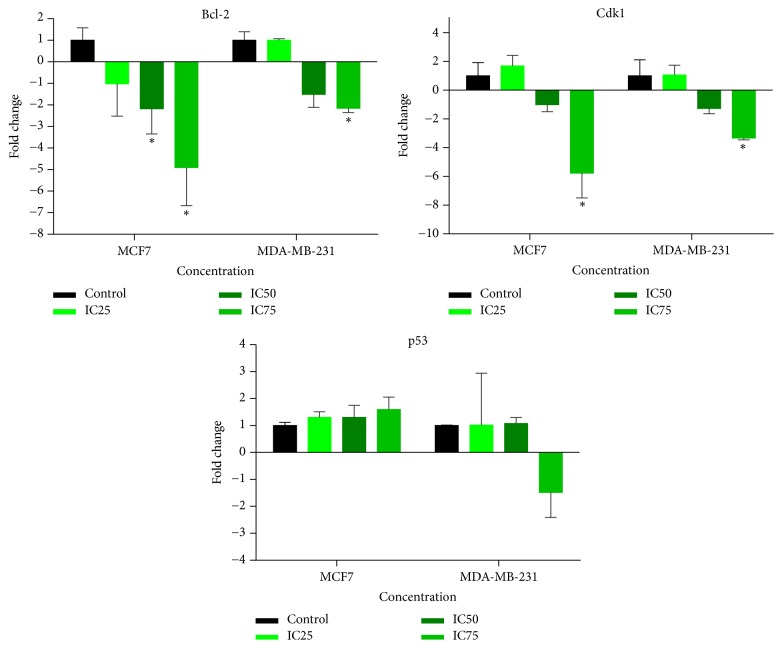
*The expression of Bcl2, Cdk1, and p53 in MCF7 and MDA-MB-231 by real-time quantitative PCR analysis*. After treatment with the IC25, IC50, and IC75 of FAA for 24 h, the relative quantification of the target gene by the delta-delta-Ct method was done using the Qiagen software. Values for the treated group are significantly different to the untreated control group at ^*∗*^*p* ≤ 0.05.

The effects of IC_25_, IC_50_, and IC_75_ value of FAA extract at 24 h on* Bcl-2*,* Cdk1*, and* p53* gene modulation were investigated by qRT-PCR. The extracted RNA from all the samples exhibited high quality and purity. The expression of* Bcl-2* gene in the treated MCF-7 cells was found to be dramatically downregulated by 2.18-fold and 4.9-fold, after treatment with IC_50_ and IC_75_ value. In MDA-MB-231 cells a significant downregulation of* Bcl-2* gene expression occurred after the treatment with IC_75_ of FAA by 2.15-fold, when compared to the untreated cells. The treatment of MCF-7 cells with IC_75_ remarkably downregulated the* Cdk1* gene expression with 5.77-fold. Moreover, the mRNA level of* Cdk1* in MDA-MB revealed a 3.33-fold downregulation at IC_75_ concentration, compared to the control. However,* p53* gene expression was found to be nonsignificant in both cell lines (Figure 6).

### 3.7. Combination Effect of FAA and Tamoxifen in MCF-7 and MDA-MB-231 Cells

**Figure 7:**
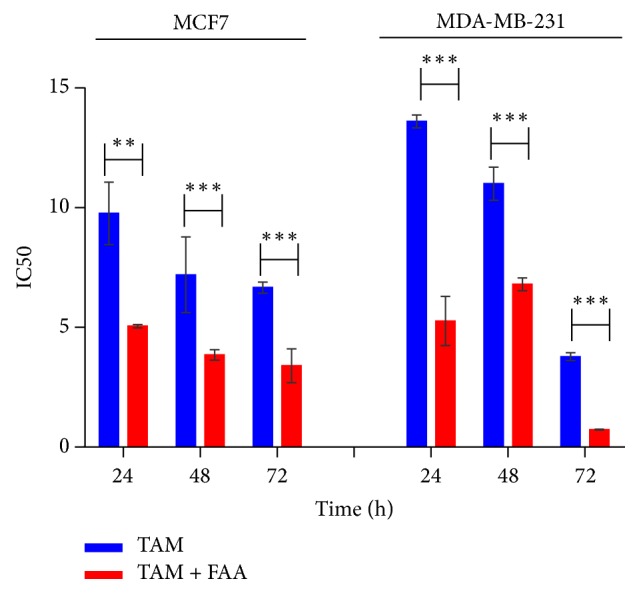
*Interaction between TAM and FAA in MCF7 and MDA-MB-231*. Cells were treated with FA-TAM in a serial dilution for 24, 48, and 72 h. Values are calculated from three independent experiments. ^*∗*^*p* < 0.05, ^*∗∗*^*p* < 0.01, and ^*∗∗∗*^*p* < 0.001 comparing the combination of TAM-FAA with TAM alone.

Based on the data showing efficacy of FAA in breast carcinoma cells, next we assessed the combinations effect of FAA and tamoxifen (TAM) on MCF-7 and MDA-MB cell growth. According to our previous study, treatment of the cells with a serial dilution of TAM, as a positive control, indicated the IC_50_ value of 9.8 *μ*g/ml, 7.2 *μ*g/ml, and 6.7 *μ*g/ml for MCF-7 and 13.6 *μ*g/ml and 11 *μ*g/ml and 3.78 *μ*g/ml for MDA-MB-231 after 24, 48, and 72 h, respectively [9]. The IC_50_ values of FAA were thus combined with the IC_50_ of TAM which resulted in more growth inhibition and less IC_50_ amount, as compared to the treatment with a single drug at 24, 48, and 72 h. Combined treatment with the IC_50_ amount of FAA-TAM reduced the IC_50_ value of MCF-7 significantly to 5.05 *μ*g/ml, 3.8 *μ*g/ml and 3.4 *μ*g/ml after 24, 48, and 72 h respectively. The IC_50_ concentration of TAM against MDA-MB-231 significantly reduced in the presence of FAA to 5.27 *μ*g/ml, 6.8 *μ*g/ml, and 0.73 *μ*g/ml at 24, 48, and 72 h, respectively (Figure 7).

**Figure 8:**
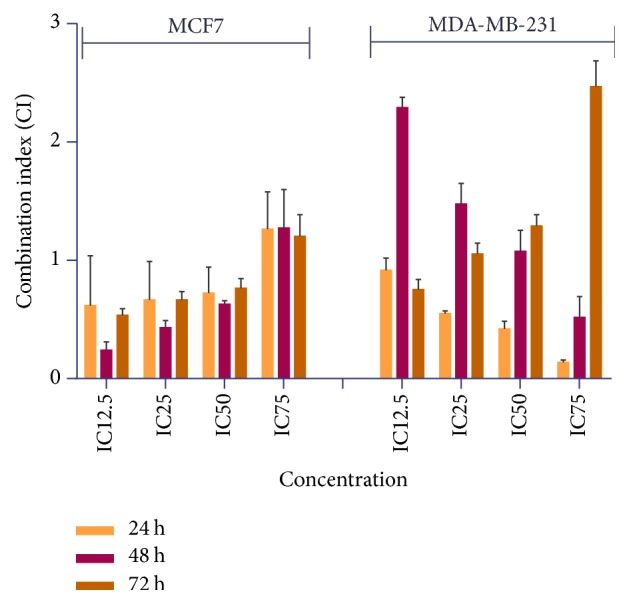
*Combination index (CI) of cotreatment with TAM and FAA on MCF7 and MDA-MB-231*. Cells were treated with FA-TAM in a serial dilution for 24, 48, and 72 h. CI value was determined by Chou and Talalay method.

Drug combination effects were analysed by the method of Chou and Talalay [13]. The varying concentrations of the combination, IC_12.5_, IC_25_, and IC_50_ concentration, demonstrated CI < 1, indicating synergistic effects, while IC_75_ showed moderate antagonism with a CI > 1. The calculated CI values for MDA-MB-231 cells treated with FAA-TAM showed a downtrend from IC_12.5_ to IC_25_, IC_50_, and IC_75_ at 24 and 48 h. The result revealed that the interaction of FAA-TAM was synergism at 24 h, even in low concentration, while only a high concentration (IC_75_) of the combination at 48 h promoted synergism but for the doses below that showed antagonism (CI > 1). Contrariwise, the CI values of FAA-TAM showed synergism was obtained only in the lowest concentration, while the higher concentration resulted in antagonism (Figure 8).

### 3.8. LC-MS Analysis of FAA Extract

**Figure 9:**
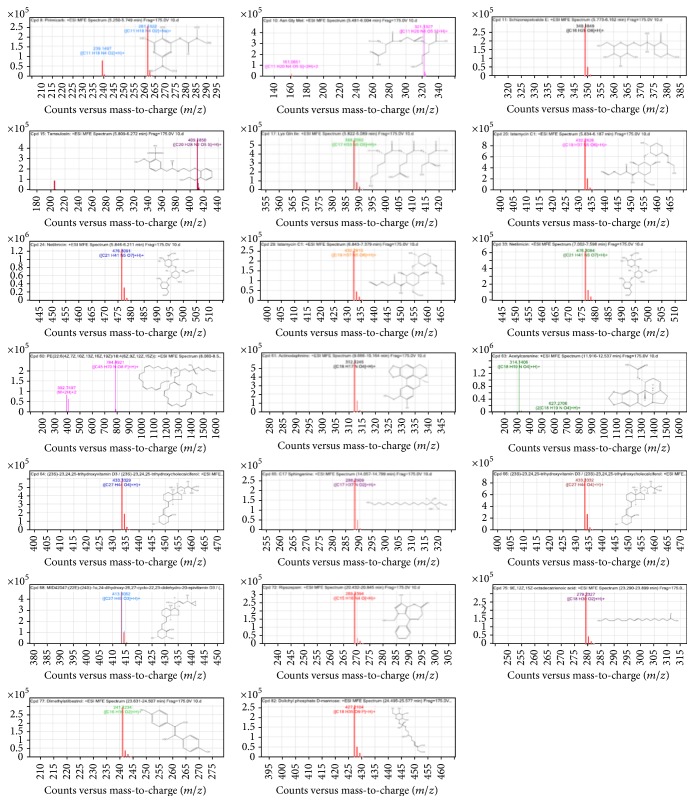
LC-MS tracings of the major compounds present in methanol extract of FAA.

Figure 9 shows LC-MS peaks for FAA and the chemical structure of major compounds while Table 1 shows the major compounds present in the extract with their molecular weight and molecular formula. Our findings indicated that the major compounds in FAA include pirimicarb, Asn Gly Met, Schizonepetoside E, tamsulosin, Lys Gln Ile, Istamycin C1, Netilmicin, Actinodaphnine, acetylcaranine, sphinganine, ripazepam, 9E,12Z,15Z-octadecatrienoic acid, dimethylstilbestrol, and dolichyl phosphate D-mannose.

## 4. Discussion

This study represents the cytotoxic activity of FAA on human breast carcinoma cancer cell lines. Here, we have shown the molecular mechanisms of apoptosis induced by FAA in MCF-7 and MDA-MB-231 breast cancer cells. Current study revealed that FAA inhibits the proliferative activity of MCF-7 and MDA-MB-231 cells. It is evident that cells exposed to FAA lose their capability to proliferate in a time- and dose-dependent manner. This finding suggests that FAA has the potential to suppress the growth of the breast cancer cells irreversibly. In addition, a dramatic reduction in the number of viable cells suggests that the antiproliferative effect of FAA may be partly due to its ability to induce cell death. Moreover, it has been reported that the FAA extract does not show toxicity towards the normal cell [10].

The selective preference is fundamentally specific targeting of tumor cells which serves to enhance the therapeutic efficacy of cytotoxic drug, while at the same time reducing organ toxicities [14]. It is highly desirable to have substances which kill the cancer cells selectively, without damaging the adjacent normal cells [15].

Analyses of FAA-treated cells by phase contrast and fluorescence microscopy are the first evidence demonstrating that morphological changes characteristic of apoptotic cell death were induced by FAA. Apoptotic cells displayed distinctive typical forms of morphological changes that are widely used for the identification of apoptosis [16, 17]. Visualization of the control (untreated) cells by phase contrast microscope showed they maintained their original morphology form. In contrast, exposure of the cancer cells to FAA for 24, 48, and 72 h exhibited classical morphologies of apoptosis, including autophagy and cell rounding due to shrinkage. After 24 h of treatment where the affected cells emerged, autophagy was achieved at maximum. In the remaining time interval, the occurrence of autophagy was not overlapped. It was caused mainly by the fact that the cell tried to gain a delay from cell death by autophagy [18], whereas, early stages of apoptosis, which are characterized by the shrinkage of cells and membrane blebbing, were clearly observed after 48 h. The increasing time of exposure to FAA causes the cells to undergo late apoptosis/necrosis. The surface changes into a big, single bubble and eventually detached [15]. The apoptosis surface morphology observed was very similar for both cell types when treated with FAA. This implied that, regardless of the induction pathway, occurrence of apoptosis is concomitant with the same morphological alterations. This is consistent with the well-known highly conserved nature of apoptosis [19].

The morphological observation by AO/PI staining in the cell nuclei of MCF-7 and MDA-MB-231 breast cancer cells showed significant morphological alterations when compared to untreated control. Most AO+ and PI– apoptotic cells were noticed with chromatin condensation staining a bright-green colour and nuclear margination, apoptotic granulation, and chromatin fragmentation with the appearances of membrane blebbing in some samples, which can be considered as moderate apoptosis. Increment of cell membrane permeability was observed after 72 h incubation, as evidenced by the apoptotic body separation and presence of reddish-orange colour due to the binding of AO to denatured DNA which can be seen clearly (AO+, PI+). Necrotic (AO–, PI+) cell populations appeared very rarely. Apoptosis and necrosis are the two major modes of cell death. Apoptosis is the more favorable mode of cell death because it is a programmed cell death mechanism that does not cause inflammatory responses. It is triggered and regulated by a variety of cellular signalling pathways. In contrast, necrotic cells are characterized by vacuolation of the cytoplasm and breakdown of plasma membrane. Due to this, the cytoplasmic elements are dispersed into the extracellular space, hence, resulting in an acute inflammatory reaction [20]. Analysis of the data disclosed that FAA had the ability to decrease the viability of MCF-7 and MDA-MB-231 cancer cells with no obvious necrotic phenomena, implying occurrence of a programmed cell death or induction of cell cycle arrest.

The cytotoxicity at the cellular level was assessed by measuring the DNA of the cancer cell in terms of distribution in cell cycle phase and ploidy level, as it flows through the cytometer [21]. Blockade of the cell cycle is considered as an effective strategy for the development of novel cancer therapies [22]. Cell cycle analysis of the treated MCF-7 revealed that FAA induced an S and G2/M phase cell cycle arrest at 24 and 48 h of treatment with an accompanying decrease in G1 phase and dose-dependent increase of sub-G0 phase. This confirmed that FAA inhibited DNA synthesis and induced a block at the S and G2/M phase boundary at 24 and 48 h, while inducing apoptosis when exposed for longer time, whereas FAA arrested cell cycle progression in MDA-MB-231 in S phase only after treatment for 72 h, concomitant with a reduction of the cells presented in G1 phase and induction of apoptosis by increasing sub-G0 phase. The major control sites included DNA damage checkpoints (G0/G1, S, and G2/M). The drug interference with initiation of DNA replication can permanently arrest the cancer cells at the S phase. Arrest in S phase requires active suppression through transactivation of suppressors, direct binding of suppressors to replication machinery, and phosphorylation of critical elements of replication control [23]. We noted that FAA damage DNA and suppress DNA replication and eventually arrest the cells in S phase. In addition, the G_2_ checkpoint allows the cell to repair DNA damage before entering mitosis [24]. In MCF-7 cells, the result of cell cycle arrest suggests that, in addition to apoptosis and S phase arrest, G2 cell cycle arrest was another factor that contributed to the preferential cell growth inhibition.

Apoptosis confirmation was carried out using the simultaneous staining of cells with FITC-Annexin-V/PI, which allows the discrimination of intact cells (FITC– PI−), early apoptotic (FITC+ PI−), late apoptotic (FITC+ PI+), and necrotic cells (FITC− PI+) via bivariate analysis [25, 26]. In the earlier events of apoptosis, membrane phospholipid phosphatidylserine (PS) is translocated from the inner to the outer leaflet of the cytoplasmic membrane and thereby exposed the PS to the external cellular environment. Translocation of PS to the outer leaflet is detectable by Annexin-V and can be quantitated using flow cytometry. Plasma membrane permeabilization converts the early apoptotic cells to late apoptotic cells. When the cytoplasmic membrane integrity is lost, the PI can penetrate to the nucleus and stain the DNA [27, 28]. Treatment with FAA exhibited that FAA induced the exposure of phosphatidylserine on the surface of MCF-7 in dose- and time-dependent manner. While in MDA-MB-231 the treatment with low and middle concentrations (IC_25_ and IC_50_) of FAA for 24 and 48 h resulted in more percentages of early apoptotic cells, the treatment for a longer time course (72 h) resulted in more cells present in the late apoptosis stage than early apoptosis. These results illustrated induction of apoptosis through multiple pathway, suggesting many compounds, rather than a single component in FAA.

Inflammation plays important roles in cancer and cancer cells produce many inflammatory mediators [29]. Although Caspases 1 and 5 play important roles in both inflammation and apoptosis [30], the role of caspases 1 and 5 in apoptosis was considered minor compared to the other caspases, in both MCF-7 and MDA-MB-231 cells. The caspase activity in treated MCF-7 revealed that FAA increased the activation of caspases 8 and 6 in all three used concentrations, while in only one concentration it increased caspase 9 (only in IC75) and caspase 3 (only in IC25) activities. The results illustrated that the apoptotic effects of FAA on MCF-7 cells were via the activation of caspase-8, which led to the activation of the final effector, caspase-6, that finally caused apoptosis. The only soluble cytosolic caspase which could cleave caspase-8 is caspase-6 [31]. In addition, caspase-6 is required for the downstream activation of caspase-8 [32]. Caspase-6 acts as an initiator caspase, by processing the initiator caspases 8 and 10, during apoptosis [33]. On the other hand, release of cytochrome* c* from mitochondria activates the downstream caspases 2 and 9 that are early biomarkers of apoptosis and eventually lead to caspase-3 activation, responsible for DNA fragmentation and morphological changes [34]. However, caspases 8 and 3 can cleave procaspase-2 without prior dimerization [35]. On the other hand, caspase-1 and caspase-5 are called inflammatory caspases, but they are also involved in the hydrolysis of procaspase-3 to the activated caspase-3 [29]. It is known that caspase-3 is a key mediator of nuclease activation that is triggered after the disruption of the mitochondrial membrane. Thus, caspase-3 activation depends on release of cytochrome* c* [36]. Therefore, this was aligned with our results, where, in the absence of caspase-1, caspase-5, caspase-2, and caspase-9 activities, the executor caspase-3 has been activated by IC_25_, probably from the other pathway.

In MDA-MB-231 cells, between the initiator caspases (caspases 2, 8, and 9), caspase-2 in IC_50_ and caspase-8 in IC_50_ and IC_75_, caspase-9 in only the IC_25_ of FAA had significantly higher activity compared to the control. Among the executioner caspases (6 and 3), only caspase-6 activity was raised in the IC_50_ of FAA. It can be concluded that, in different concentrations, various caspases are activated. In the IC_50_ concentration of FAA, initiator caspases 2 and 8 resulted in the activation of caspases 6 and 3, respectively. In IC_25_ and IC_75_ concentrations of FAA, none of the executioner caspases 3 or 6 was activated, while caspase-9 and caspase-8 were activated in at least one of these concentrations. Firstly, the results gave the possibility that apoptosis in MDA-MB-231 cells in these concentrations was not going through the caspase activation pathway. More and more studies have illustrated that apoptosis is not synonymous with the activation of caspases [37]. Secondly, maybe the executioner caspase-7 was activated in these concentrations. Both caspases 3 and 7 could be activated by caspases 8 and 9 during apoptosis. Caspases 3 and 7 have overlapping, but also distinct roles in apoptosis that is cell type and stimulus dependent [38].

*Bcl-2* family of genes plays a critical role in the regulation of the apoptotic process initiated at the mitochondria [39] and governed the mitochondrial outer membrane permeabilization [40]. Given that FAA has been identified as inducers of apoptosis, we sought to detect the* Bcl-2* mRNA level in MCF-7 and MDA-MB-231 after treatment with FAA. The expression of* Bcl-2* exhibited an initial strong decrease, illustrating the potential of FAA to affect the transcriptional regulation of* Bcl-2* and the degradation of its mRNA. Assessment of* Bcl-2* levels in both cells upon treatment by FAA also demonstrated some significant modulations, depending on the initial dose of exposure. The increment of FAA concentrations from IC_25_ to IC_75_ in MCF-7 cells showed a downward trend of* Bcl-2* expression. On the other hand, MDA-MB-231 displayed a dramatic downregulation of* Bcl-2* in IC_75_ of FAA. The mechanism of apoptosis inhibition by* Bcl-2* is through the suppression of caspase proteins [41]. Caspase-9 induces loss of mitochondrion membrane concomitant with the* Bcl-2* and* Bcl-xL* cleavage and therefore inactivates the antiapoptotic functions of these molecules and will potentially disrupt the balance between pro- and antiapoptotic molecules and induce cell death [42]. The* Bcl-2* expression observed in MCF-7 cells was aligned with the activity level of caspase-9, since downregulation of* Bcl-2* was concomitant with caspase-9 activity. In contrast, the result of MDA-MB-231, which was in an argument with the observation of caspase-9 activity, demonstrated that FAA lead to* Bcl-2* downregulation, without impairing the mitochondrial functions which confirm the extramitochondrial roles of* Bcl-2*.

Cell cycle regulatory proteins had been considered as predictive markers in cancers. Cyclin-dependent protein kinases (*Cdk*s) are key signalling molecules in the regulation of cell cycle [43] for entry into mitosis and a core component in spindle morphogenesis [44].* Cdk1* is the only necessary* Cdk* in the process of cell proliferation through mitosis. As* Cdk1* inhibitors effectively arrested tumor cell growth, finding the new* Cdk1* inhibitors is a new target in the research and development of anticancer drugs [45, 46]. It has been evident that* Cdk1*, which is overexpressed and have enhanced kinase activity in many tumor types, is the potential targets for cancer therapy [47]. FAA extract succeeded in reducing the expression of* Cdk1* in both cells in a dose-dependent manner, as IC_75_ showed the significantly lower expression in MCF-7 and MDA-MB-231. The inhibition of G2/M progression induced by FAA in MCF-7 suggests that the* Cdk1*-cyclin B complex regulates the G2 to M transition and resulted in the antiproliferative effect of FAA. In contrast, although downregulation of* Cdk1* occurred in MDA-MB-231, only sub-G0/G1 phase arrest was observed (in the same concentration at 24 h) that demonstrated apoptosis in this cell line. Since* Cdk1* transcription is controlled by various factors, we hypothesize that treatment with FAA in MDA-MB-231 may affect* Cdk1* transcription via inhibition of protein kinases involved in the upstream regulation of this gene [48]. Moreover, it has been shown that* Cdk1* may be required for apoptosis, independent of the cell cycle regulation [49].

The* p53* tumor suppressor gene includes various functional categories and plays a role in repair of DNA damage, cell cycle progression, and apoptosis [50]. The activation of specific* p53* response could be determined by the specific transcriptional targets and roles in subcellular pathways [51]. In the current study,* p53* expression in MCF-7 and MDA-MB-231 was not significantly upregulated. The current study did not exclude the possibility that some of the cells that were negative for nuclear* p53* expression may have a* p53* gene mutation [52]. About 50% of all cancer cases possess a* p53* mutation with loss of its tumor suppressor activities; therefore these cases are resistant to apoptosis and required new drugs that act in a* p53*-independent pathways [53]. The novel plant extract FAA demonstrated the ability to kill breast cancer cells by a* p53*-independent mechanism, while it was associated with extrinsic or intrinsic signalling pathway and downregulation of* Bcl-2*, depending on the concentrations.

Since cancer is the result of numerous mutations reposition, combination of two or more drugs with different mechanisms of action may lead to enhancing cell death [54]. FAA could be useful in synergizing the efficacy of TAM in both MCF-7 and MDA-MB231 cells. The significance of this finding lies in the fact that, besides its therapeutic potency, TAM cause high toxicity to the normal tissues and harmful effects on health and development of primary and secondary drug resistance limiting its clinical success in the cancer chemotherapy. In this regard, current report shows that combination of chemotherapeutic agents with naturally occurring dietary supplements is a preferable modality for cancer treatment than single-agent that might also reduce systemic toxicity of the chemotherapeutic agents [55].

LC-MS was utilized to separate and evaluate chemical constituents in FAA which indicated the presence of different phytochemicals in the respective extract with various biological actions such as antioxidant and anticancer activates. For instance, sulfonamide derivatives tamsulosin reduces prostate smooth muscle tone and thereby inhibits the dynamic component of the obstruction, utilized in prostate cancer [56]. Actinodaphnine, an aporphine alkaloid, has been shown to possess antitopoisomerases, antibacterial, antifungal, and anticancer properties. It also possesses antiplatelet aggregation and vasorelaxant activity [57]. Acetylcarnitine is an acetylated form of carnitine which has antioxidant activities through direct scavenging free radicals and chelating catalytic metals-promoters of reactive oxidative species (ROS) [58]. Trihydroxyvitamin D3 is a product of vitamin D3 metabolism; several vitamin D analogs are entering clinical trials and are showing great promise as potential therapeutic agents for the treatment of cancer and other diseases [59]. Sphingoid bases may function as chemotherapeutic as well as chemopreventive agents by preferentially inhibiting cancer cells and eliminating stem cells from which most breast cancer cells arise [60]. 9,12,15-Octadecatrienoic acid is another major compound in the studied plant extracts and has been reported to have antimicrobial, antioxidant, and anti-inflammatory activities [61]. The results show that the occurrence of the variable compounds may be responsible for the different extent of anticancer activity shown by the test extract.

## 5. Conclusion

In conclusion, FAA inhibited cell proliferation MCF-7 and MDA-MB-231 cells through stimulation of cell-specific S and G2/M arrest in MCF-7 and S cell cycle phase arrest in MDA-MB-231 accompanied by induction of apoptosis through both extrinsic and intrinsic apoptotic pathways, probably in the result of biological properties of phytochemicals present in FAA extract. In addition, the FAA-induced apoptosis in both cells is likely to be caspase-dependent,* Bcl-2*-dependent, and* p53*-transcription-independent. FAA also exhibited synergistic effect when combined with TAM, suggesting that it can complement current chemotherapeutic treatment.

## Supplementary Material

I. Representative DNA frequency histograms of MCF7 without treatment (A), treatment with IC25 of FAA (B), IC50 of FAA (C), IC75 of FAA (D) for 24 h; without treatment (A′), treatment with IC25 of FAA (B′), IC50 of FAA (C′), IC75 of FAA (D′) after 48h and without treatment (A′′), treatment with IC25 of FAA (B′′), IC50 of FAA (C′′), IC75 of FAA (D′′) after 72 h, analyzed by flow cytometer. II. Representative DNA frequency histograms of MDA-MB-231 without treatment (A), treatment with IC25 of FAA (B), IC50 of FAA (C) , IC75 of FAA (D) for 24 h, without treatment (A′), treatment with IC25 of FAA (B′), IC50 of FAA (C′) , IC75 of FAA (D′) for 48 h and without treatment (A′′), treatment with IC25 of FAA (B′′), IC50 of FAA (C′′) , IC75 of FAA (D′′) for 72 h; analyzed by flow cytometer.

## Figures and Tables

**Table 1:** LC-MS characterization of methanolic flower extract of *Allium atroviolaceum*.

Peak number	Rt	Mass	Formula	Compound name
8	5.485	238.143	C_11_H_18_N_4_O_2_	Pirimicarb
10	5.662	320.1157	C_11_H_20_N_4_O_5_S	Asn Gly Met
11	5.848	348.1775	C_16_H_28_O_8_	Schizonepetoside E
15	5.9	408.1718	C_20_H_28_N_2_O_5_S	Tamsulosin
17	5.915	387.2487	C_17_H_33_N_5_O_5_	Lys Gln Ile
20	5.95	431.2753	C_19_H_37_N_5_O_6_	Istamycin C1
24	5.989	475.3017	C_21_H_41_N_5_O_7_	Netilmicin
60	8.339	783.4845	C_45_H_70_NO_8_P	PE(22:6(4Z,7Z,10Z,13Z,16Z,19Z)/18:4(6Z,9Z,12Z,15Z))
61	9.846	311.1171	C_18_H_17_NO_4_	Actinodaphnine
63	12.087	313.131	C_18_H_19_NO_4_	Acetylcaranine
64	14.88	432.3255	C_27_H_44_O_4_	(23S)-23,24,25- trihydroxyvitamin D3/(23S)- 23,24,25- trihydroxycholecalciferol
65	14.417	287.2835	C_17_H_37_NO_2_	C17 sphinganine
68	18.315	412.2989	C_27_H_40_O_3_	MID42047:(22E)-(24S)-1*α*,24- dihydroxy-26,27-cyclo-22,23- didehydro-20-epivitamin D3/(22E)-(24S)-1*α*,2
72	20.701	268.1322	C_15_H_16_N_4_O	Ripazepam
75	23.633	278.2254	C_18_H_30_O_2_	9E,12Z,15Z-octadecatrienoic acid
77	23.946	240.1161	C_16_H_16_O_2_	Dimethylstilbestrol
82	25.069	426.2032	C_18_H_35_O_9_P	Dolichyl phosphate D-mannose
