# Supplementary material for: Cytotoxicity and Proapoptotic Effects of Allium atroviolaceum Flower Extract by Modulating Cell Cycle Arrest and Caspase-Dependent and p53-Independent Pathway in Breast Cancer Cell Lines
Source: Evid Based Complement Alternat Med. 2017 Nov 8;2017:1468957. doi: 10.1155/2017/1468957 (PMC5698829; doi:10.1155/2017/1468957)
Supplement: Supplementary file 1 — I. Representative DNA frequency histograms of MCF7 without treatment (A), treatment with IC25 of FAA (B), IC50 of FAA (C), IC75 of FAA (D) for 24 h; without treatment (A′), treatment with IC25 of FAA (B′), IC50 of FAA (C′), IC75 of FAA (D′) after 48h and without treatment (A′′), treatment with IC25 of FAA (B′′), IC50 of FAA (C′′), IC75 of FAA (D′′) after 72 h, analyzed by flow cytometer. II. Representative DNA frequency histograms of MDA-MB-231 without treatment (A), treatment with IC25 of FAA (B), IC50 of FAA (C) , IC75 of FAA (D) for 24 h, without treatment (A′), treatment with IC25 of FAA (B′), IC50 of FAA (C′) , IC75 of FAA (D′) for 48 h and without treatment (A′′), treatment with IC25 of FAA (B′′), IC50 of FAA (C′′) , IC75 of FAA (D′′) for 72 h; analyzed by flow cytometer. [file 1468957.f1.pdf]

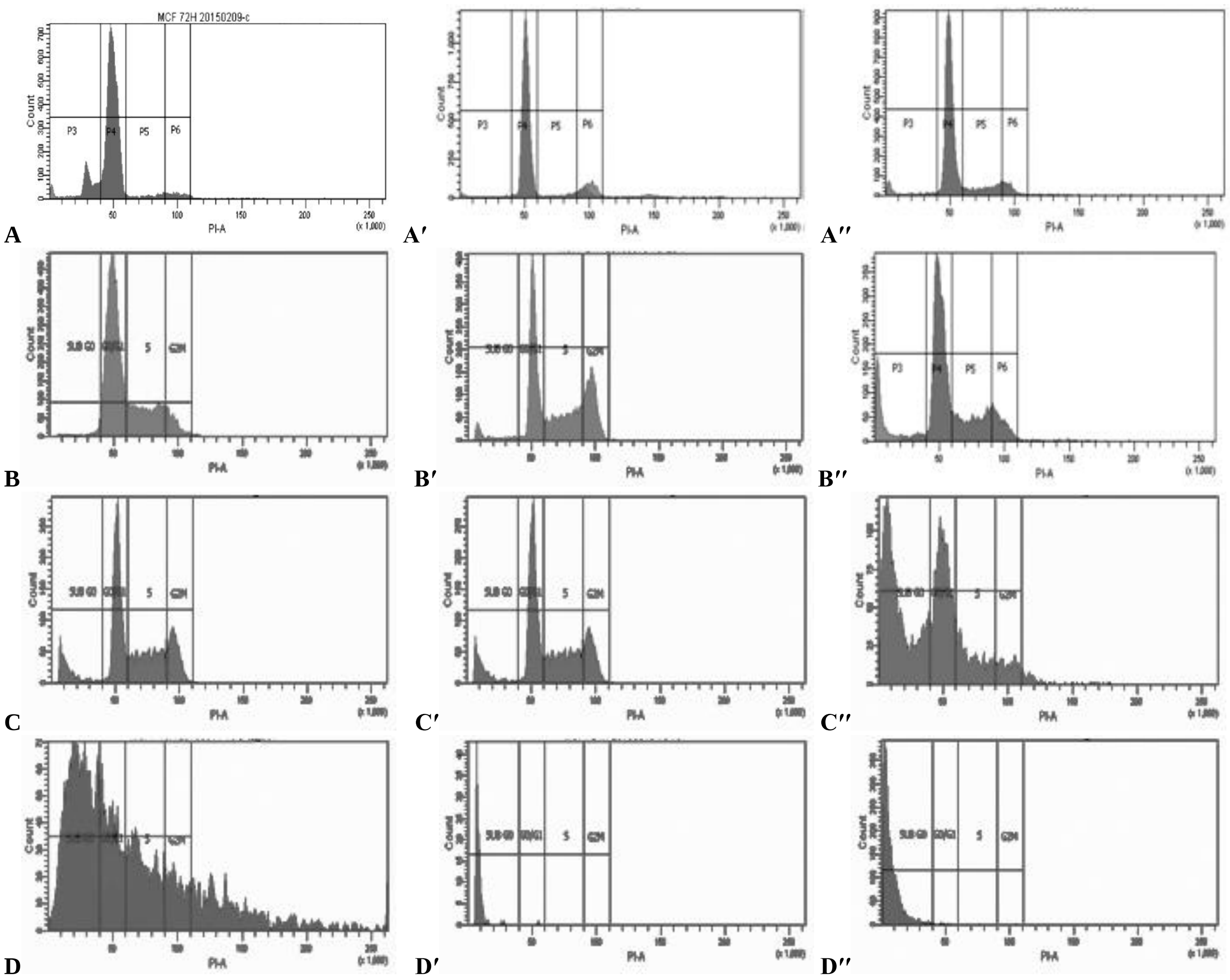

I. Representative DNA frequency histograms of MCF7 without treatment (A), treatment with IC_{25} of FAA (B), IC_{50} of FAA (C), IC_{75} of FAA (D) for 24 h; without treatment (A′), treatment with IC_{25} of FAA (B′), IC_{50} of FAA (C′), IC_{75} of FAA (D′) after 48h and without treatment (A″), treatment with IC_{25} of FAA (B″), IC_{50} of FAA (C″), IC_{75} of FAA (D″) after 72 h, analyzed by flow cytometer.

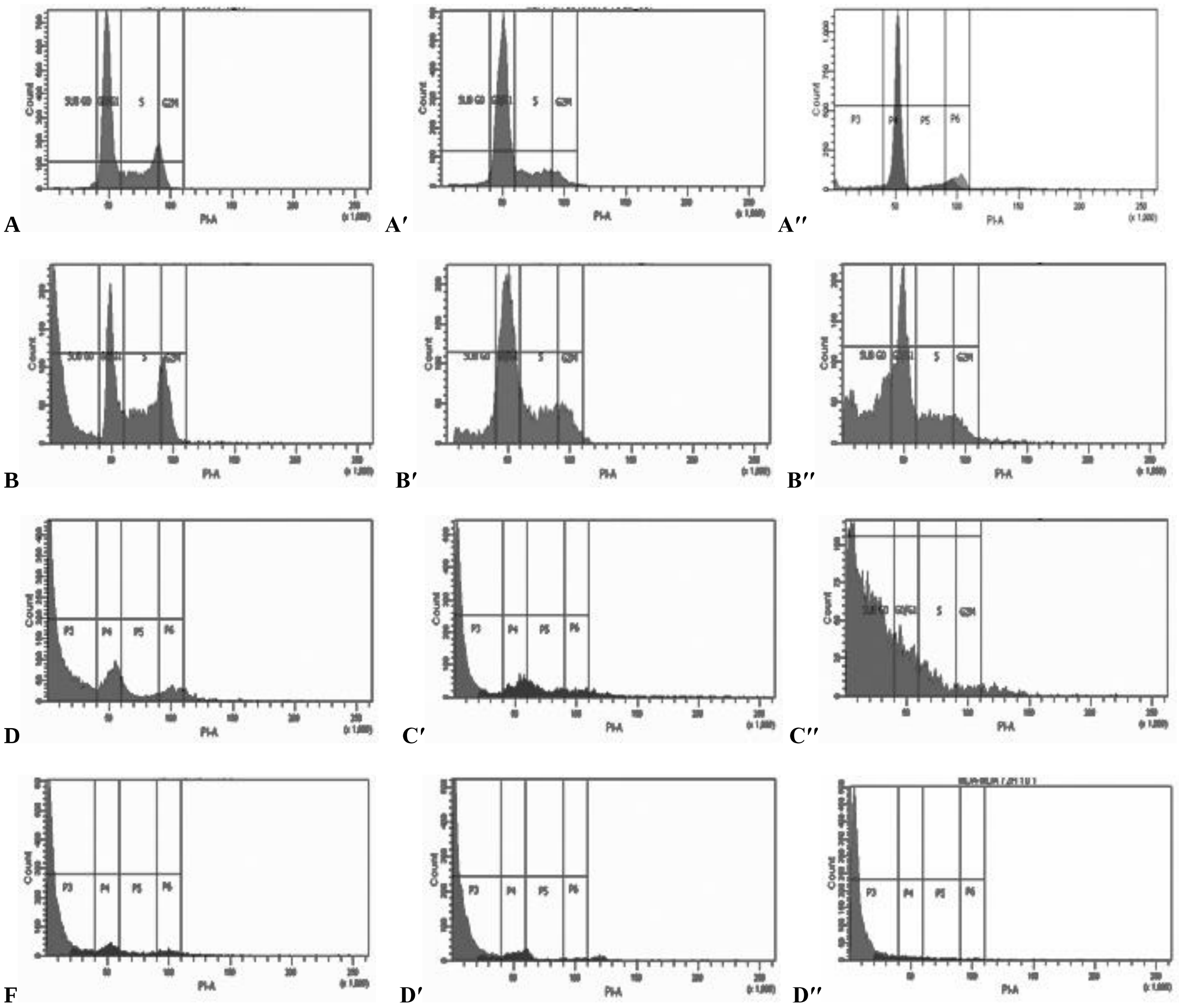

II. Representative DNA frequency histograms of MDA-MB-231 without treatment (A), treatment with IC_{25} of FAA (B), IC_{50} of FAA (C) , IC_{75} of FAA (D) for 24 h, without treatment (A′), treatment with IC_{25} of FAA (B′), IC_{50} of FAA (C′) , IC_{75} of FAA (D′) for 48 h and without treatment (A″), treatment with IC_{25} of FAA (B″), IC_{50} of FAA (C″) , IC_{75} of FAA (D″) for 72 h; analyzed by flow cytometer.
